# Identifying the Clusters within Nonmotor Manifestations in Early Parkinson's Disease by Using Unsupervised Cluster Analysis

**DOI:** 10.1371/journal.pone.0091906

**Published:** 2014-03-18

**Authors:** Hui-Jun Yang, Young Eun Kim, Ji Young Yun, Han-Joon Kim, Beom Seok Jeon

**Affiliations:** 1 Department of Neurology, Ulsan University Hospital, Ulsan, Korea; 2 Department of Neurology and Movement Disorder Center, Parkinson Study Group, Neuroscience Research Institute, Seoul National University Hospital, Seoul, Korea; 3 Department of Neurology, Ewha Womans University Mokdong Hospital, Seoul, Korea; Hospital General Dr. Manuel Gea González, Mexico

## Abstract

**Background:**

Classical and data-driven classifications of Parkinson's disease (PD) are based primarily on motor symptoms, with little attention being paid to the clustering of nonmotor manifestations.

**Methods:**

Clinical data on demographic, motor and nonmotor features, including the Korean version of the sniffin' stick (KVSS) test results, and responses to the screening questionnaire of the nonmotor features were collected from 56 PD patients with disease onset within 3 years. Nonmotor subgroups were classified using unsupervised hierarchical cluster analysis (HCA). In addition to unsupervised HCA, we performed a cross-sectional analysis comparing the performance on the KVSS olfactory test with other nonmotor manifestations of the patients.

**Results:**

Forty-nine patients (87.5%) had hyposmia based on the KVSS test. HCA suggested three nonmotor clusters for all PD patients and two nonmotor clusters in *de novo* PD patients, without *a priori* assumptions about the relatedness. In the cross-sectional analysis, dream-enactment behavior was more prevalent in patients with lower olfactory scores, implying impaired olfactory function (*P* = 0.029 for all PD patients; *P* = 0.046 for *de novo* PD patients).

**Conclusion:**

We propose the existence of different clusters of nonmotor manifestations in early PD by using unsupervised hierarchical clustering. To our knowledge, this study is the first to report the identification of nonmotor subgroups based on unsupervised HCA of multiple nonmotor manifestations in the early stage of the disease.

## Introduction

Parkinson's disease (PD) is a clinically and pathologically heterogeneous disease [1]. This heterogeneity is thought to indicate different subtypes of PD and many studies have sought to elucidate them [2], [3].

Classically, patients are divided by their motor phenotype into tremor-dominant (TD) or postural instability-gait disturbance (PIGD) subgroups, or classified by onset age as juvenile, young, or late onset PD [1]. In recent years, cluster analysis has been introduced as an objective data-driven grouping method [2], [4]. Several cluster analysis studies have shown that the age of onset [4]–[9], rate of disease progression [5]–[10], and motor phenotype [6], [9]–[10] are the major dimensions of PD subgroup classification.

The classical and data-driven approaches have focused primarily on the motor symptoms of PD. Some of the nonmotor features, such as depression [8] or cognitive decline [4], [10] were associated with previous classifications based mainly on motor phenotypes. Although recent results from two independent European cohorts have shown that the severity of nonmotor symptoms as well as motor complications are important factors in the characterization of PD subtypes [11], little attention has been paid to the clustering of nonmotor manifestations themselves [12], [13].

In the current study, we investigated the existence of different subgroups of nonmotor manifestations by using unsupervised hierarchical cluster analysis (HCA). To our knowledge, this is the first identification of nonmotor clusters using unsupervised HCA on a range of nonmotor features in early stage PD.

## Methods

### Subjects

Between July 2007 and January 2008, 119 consecutive patients with idiopathic PD who met the diagnostic criteria of the United Kingdom Brain Bank were referred to our movement disorder center and clinically followed up. At referral, 56 of these patients who were within 3 years of motor symptom onset underwent olfactory function testing using the Korean version of the sniffin' stick (KVSS) test, the first olfactory function test to use odorants familiar to Koreans [14]; moreover, these patients were personally interviewed by a trained member of the movement disorders clinic by utilizing a screening questionnaire consisting of 8 nonmotor items in Korean, as part of the routine clinical evaluation of patients with PD. Patients were asked about the presence or absence of each nonmotor feature (insomnia, orthostatic dizziness, depression, excessive daytime sleepiness, urinary symptom, memory disturbance, and dream-enactment behavior) mentioned in each item.

We retrospectively performed a systematic review of the hospital electronic medical records to collect clinical data on current age, age at onset of PD, gender, Hoehn and Yahr (H-Y) stage, dopaminergic drugs in a levodopa-equivalent daily dose (LEDD, mg/day), presence of constipation and responses to the screening questionnaire of the nonmotor features. Constipation was defined as having fewer than three bowel movements per week. Patients had to be followed for at least 1 year to be included. None of the *de novo* PD patients, a subset of the all PD samples, had previously taken antiparkinsonian medication. This retrospective study was approved by the Institutional Review Board (IRB) at the Seoul National University Hospital (H-1109-122-379). Requirement for informed consent was waived for this retrospective analysis of clinical data. The IRB also approved the consent procedure. All data used in this study was analyzed anonymously.

### Smell testing

The KVSS test is a modified version of the “Sniffin' Stick” test [14]. Its validity and reliability have been demonstrated in comparison with the Cross-cultural Smell Identification Test (CC-SIT) [15]. KVSS I is a rapid screening test and KVSS II is a comprehensive test that involves three subsets: threshold, discrimination, and identification. The olfactory threshold was defined as the mean concentration at which the pen containing *n*-butanol was differentiated correctly four times from two blank pens. Olfactory discrimination was assessed using triplets of odorant pens in which two pens have identical odors and the other has a different odor, and the patients identify the pen with a different odorant. For olfactory identification, all 16 different odorants familiar to Koreans were presented in felt-tip pens, with the patients choosing one of four odor items [14]. The scores ranged from 0 to 16 in all three subsets. The sum of the threshold, discrimination, and identification subset scores is presented as the composite threshold-discrimination-identification (TDI) score.

### Statistical analysis

Statistical analyses were performed with the open-source statistical software R version 2.15.1 (http://www.r-project.org) and IBM SPSS statistics version 19.0 (IBM, Somers, NY).

Unsupervised HCA was performed and the clustering results are shown using a dendrogram. The main advantage of an unsupervised hierarchical approach is that it can be applied when the optimum number of clusters is not known in advance [16]. We used Yule's Q as a measure of similarity for asymmetric binary variables. The dissimilarity between clusters was calculated by the most common method, unweighted pair group method with arithmetic mean (UPGMA) also known as between-group average method [17]. Different methods of measuring similarity (Jaccard's coefficient, Dice's coefficient and Yule's Q) and different measures of intergroup distance (average linkage within-groups method, and UPGMA) were used to demonstrate the stability of the clustering [16], [17]. Multiscale bootstrap resampling was performed with the R package pvclust version 1.2–2 to compute the confidence of the hierarchical clustering with 1000 bootstrapped samples [18], [19]. Clusters with approximately unbiased probability value (AU *P* value) >95% were considered significant [19].

The cross-sectional analysis between the two olfactory groups based on the performance in the KVSS test was performed independently with unsupervised cluster analysis. The patients were divided into high- and low-scoring groups by using the KVSS II median TDI score along previous literatures [20]. The chi-square test and Fisher's exact test were used to determine the relationship between olfactory dysfunction and other nonmotor manifestations. Either Student's *t*-test or Mann-Whitney *U* test was used to analyze clinical differences between the two olfactory groups. The results were considered statistically significant at *P*<0.05.

## Results

**Figure 1:**
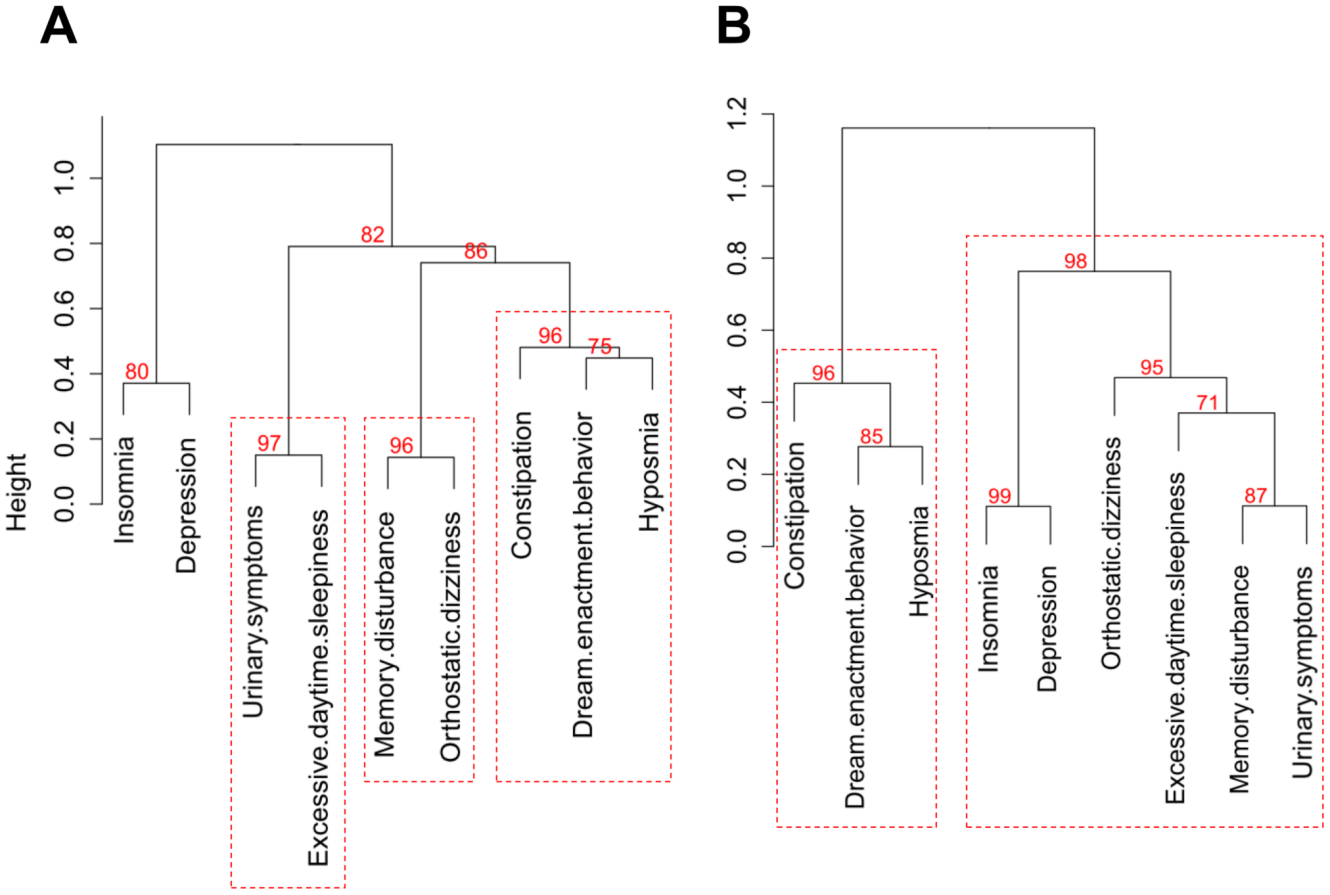
Dendrogram of the hierarchical cluster analysis and multiscale bootstrap resampling results of nonmotor features of Parkinson's disease (PD). The clusters with an approximately unbiased (AU, red) *P*-value>95% are highlighted by the red dashed rectangles. (**A**) all PD patients. (**B**) *de novo* PD patients.

Table 1 shows demographic and clinical characteristics of the subjects. The 56 patients included 28 men and 28 women (age range, 46–81 years). No subject had motor fluctuation or levodopa-induced dyskinesia. Twenty-seven patients were newly diagnosed *de novo* PD patients. The KVSS tests were well accepted by all patients. Forty-nine patients (87.5%) had hyposmia based on the reported criteria [14], [15]. For all of the PD patients, the cluster analysis of nine nonmotor features gave three clusters without *a priori* assumptions about relatedness. Figure 1A shows the corresponding dendrogram based on UPGMA distance. Cluster 1 included hyposmia, dream-enactment behavior, and constipation. Cluster 2 comprised memory disturbance and orthostatic dizziness. Cluster 3 contained urinary symptoms and excessive daytime sleepiness.

**Table 1:** Demographics and clinical profiles of the PD patients.

	All PD patients (n = 56)							*de novo* PD patients (n = 27)						
Characteristics	Total		TDI top (n = 28)		TDI bottom (n = 28)		*P*	Total		TDI top (n = 14)		TDI bottom (n = 13)		*P*
Age (years)	64.0	(8.5)	62.1	(9.9)	65.9	(6.5)	.101	64.5	(7.7)	64.4	(8.2)	64.7	(7.4)	.912
Gender (% male)	50.0		42.9		57.1		.285	48.1		42.9		53.8		.568
Age at PD onset (years)	62.6	(8.8)	60.7	(10.5)	64.5	(6.4)	.103	63.6	(8.0)	63.4	(8.5)	63.7	(7.8)	.934
Duration of PD (years)	1.6	(0.9)	1.5	(1.0)	1.6	(1.0)	.871	1.3	(0.8)	1.2	(0.7)	1.4	(0.9)	.450
Hoehn and Yahr stage	2.0	(0.7)	2.0	(0.7)	2.1	(0.7)	.618	2.1	(0.7)	2.1	(0.7)	2.0	(0.7)	.939
I (%)	16	(28.6)	8	(28.6)	8	(28.6)		6	(22.2)	3	(21.4)	3	(23.1)	
II (%)	30	(53.6)	15	(53.6)	15	(53.6)		16	(59.2)	8	(42.9)	8	(38.5)	
III (%)	10	(17.9)	5	(17.9)	5	(17.9)		5	(18.5)	3	(21.4)	2	(15.4)	
LEDD, mg/day	149.6	(231.1)	101.2	(188.1)	198.1	(261.8)	.118							
KVSS I	5.0	(1.5)	5.7	(0.7)	4.1	(1.4)	<0.001	5.3	(1.2)	5.9	(0.7)	.4.7	(1.3)	.009
KVSS II thresholds	4.1	(3.5)	6.2	(3.3)	2.0	(2.1)	<0.001	4.6	(3.8)	7.1	(3.5)	1.8	(1.5)	<0.001
KVSS II discrimination	8.0	(2.6)	9.6	(1.9)	6.4	(2.3)	<0.001	8.3	(3.3)	9.9	(2.4)	6.6	(3.3)	.009
KVSS II identification	8.2	(2.7)	10.1	(2.2)	6.3	(1.6)	<0.001	8.7	(3.1)	10.7	(2.7)	6.6	(1.9)	<0.001
TDI score	20.1	(6.6)	25.5	(3.4)	14.7	(4.1)	<0.001	21.3	(7.4)	27.1	(2.4)	15.0	(5.6)	<0.001

Data are shown as means (standard deviation) unless otherwise indicated. *P*-values are from Student's *t*-test for continuous measures or Mann-Whitney *U* test for ordinal data. PD, Parkinson's disease; LEDD, levodopa equivalent daily dose; KVSS, Korean version of the sniffin' stick; TDI, threshold-discrimination-identification.

HCA in the *de novo* PD group revealed two main clusters (Fig. 1B). Cluster 1 was defined by hyposmia, dream-enactment behavior, and constipation. The larger cluster 2 was defined by depression, insomnia, memory disturbance, orthostatic dizziness, excessive daytime sleepiness, and urinary symptoms. The clustering stability was assessed by comparing the results of different methods of measuring similarity and different measures of intergroup dissimilarity. We obtained similar clustering results and concluded that the group structure was stable.

**Figure 2:**
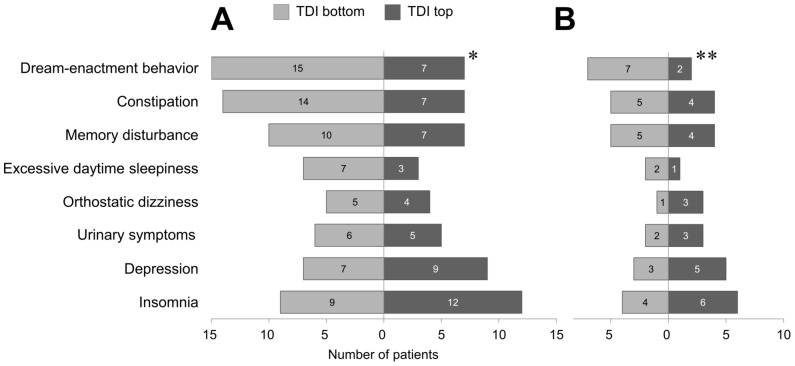
Group differences in nonmotor manifestations based on the KVSS II TDI score (*P = 0.029; **P = 0.046, chi-square test or Fisher's exact test, as appropriate). (**A**) all PD patients. (**B**) *de novo* PD patients.

In cross-sectional investigation, the patients were split into two olfactory groups based on the median TDI score [20]. Gender distribution, mean current age, disease duration, LEDD, and H-Y stage did not differ between the two olfactory groups (Table 1). Dream-enactment behavior was more prevalent in patients with lower TDI scores, which imply impaired olfactory function (*P* = 0.029 for all PD patients; *P* = 0.046 for *de novo* PD patients; Fig. 2). There were no significant differences in other nonmotor symptoms (Figs. 2A and 2B).

## Discussion

In this study, 87.5% of patients were hyposmic as assessed by the KVSS olfactory test, which was in line with results from previous olfactory function studies in PD patients [18], [20], [21]. Unsupervised cluster analysis suggests three nonmotor clusters for the entire group of PD patients and two clusters for the *de novo* PD patients. This clustering result and the cross-sectional investigation result are in agreement with results of previous studies demonstrating close relationships between olfactory dysfunction and rapid eye movement sleep behavior disorders (RBDs) [22]. Notably, our study suggests that depression is independent of olfactory dysfunction. This is a somewhat striking result, since depression, together with hyposmia, is a well-known predated nonmotor manifestation [23]. Our clustering result concurs with those of several works showing that olfactory dysfunction did not correlate with depression [20], [24], [25]. The discrepancy with the findings of Berendse *et al.*, which addressed a potential correlation of hyposmia with depression, might partly depend on disease duration or the use of different olfactory function tests [26].

Some nonmotor manifestations, such as hyposmia, RBDs, constipation, and depression, can develop during the prodromal period of PD and precede the onset of classical PD motor symptoms [27], [28]. While patients in the premotor stage of PD exhibit no motor symptoms, they have do demonstrate various prodromal nonmotor symptoms [23], [29]. Conventional PD classification based on motor phenotype (tremor-dominant or PIDG) cannot be applied to these premotor PD; therefore, classifications that are wholly based on nonmotor symptoms are needed. The finding of this study can have implications for developing the premotor PD classification based on nonmotor features. Our research subjects, patients with newly diagnosed *de novo* PD and patients with relatively early stages of PD (with disease onset within 3 years), may be the closest diagnostic PD group to represent ideal premotor PD populations. Our study demonstrates the existence of different nonmotor symptom clusters in early stage PD patients and support the idea of a premotor PD subtype based on nonmotor manifestations [30]–[32].

While this cluster analysis gave interesting results, the clinical relevance of the complicated relationships among nonmotor manifestations is yet to be determined. A nonmotor feature could be caused by coexisting conditions, for example, daytime sleepiness due to nocturia. It is also possible that some symptoms are caused by PD medication, such as levodopa or dopamine agonists [33]. In addition, the patterning of nonmotor manifestations seen in our results implicates common neuropathological and neurochemical processes underlying PD [13], [34], [35]. The underlying alpha-synuclein pathology has been proposed as a shared mechanism for hyposmia, RBD, and constipation [22], [35], which define the first cluster of *de novo* PD patients (Fig. 1B). Other nonmotor features that comprise the second cluster of *de novo* PD patients, such as depression, memory disturbance and autonomic dysfunction, may be early disease manifestations that reflect underlying non-dopaminergic deficit [23], [36].

One limitation of this study is its retrospective design with a relatively small sample size, which potentially influences the generalizability of our results. Another limitation is the use of the screening questionnaire of the nonmotor features that has not been validated. The lack of replication is another caveat of this study. Although we used multiscale bootstrap resampling to validate confidence in the clusters, a larger study should be conducted to verify our results [2], [18].

This preliminary analysis examining the presentation of multiple nonmotor features suggests that it is possible to identify structures based on the profiles of nonmotor manifestations. Future data-driven replications with larger independent populations are required, and these will serve to increase our understanding of associations between various nonmotor features of PD.
